# Estrogen Replacement Reduces Oxidative Stress in the Rostral Ventrolateral Medulla of Ovariectomized Rats

**DOI:** 10.1155/2016/2158971

**Published:** 2015-11-10

**Authors:** Fan Hao, Ying Gu, Xing Tan, Yu Deng, Zhao-Tang Wu, Ming-Juan Xu, Wei-Zhong Wang

**Affiliations:** ^1^Department of Obstetrics and Gynecology, Changhai Hospital, Second Military Medical University, Shanghai 200433, China; ^2^Department of Physiology, Second Military Medical University, Shanghai 200433, China

## Abstract

Cardiovascular disease prevalence rises rapidly after menopause, which is believed to be derived from the loss of estrogen. It is reported that sympathetic tone is increased in postmenopause. The high level of oxidative stress in the rostral ventrolateral medulla (RVLM) contributes to increased sympathetic outflow. The focus of this study was to determine if estrogen replacement reduces oxidative stress in the RVLM and sympathetic outflow in the ovariectomized (OVX) rats. The data of this study showed that OVX rat increased oxidative stress in the RVLM and sympathetic tone; estrogen replacement improved cardiovascular functions but also reduced the level of oxidative stress in the RVLM. These findings suggest that estrogen replacement decreases blood pressure and sympathoexcitation in the OVX rats, which may be associated with suppression in oxidative stress in the RVLM through downregulation of protein expression of NADPHase (NOX4) and upregulation of protein expression of SOD1. The data from this study is beneficial for our understanding of the mechanism of estrogen exerting cardiovascular protective effects on postmenopause.

## 1. Introduction

The prevalence and severity of cardiovascular diseases (e.g., hypertension and coronary heart disease) increase more markedly along with increasing age in postmenopausal women. A higher percentage of women than men suffer from hypertension after the age of 65 years [1, 2]. It has been indicated that estrogen possesses beneficial effects on these cardiovascular diseases [3]. Beside its well-known peripheral cardiovascular protective effects, accumulating evidence shows that estrogen is recognized as a modulator in the central nervous system (CNS) to cardiovascular regulation. For example, activation of estrogen receptors in the cardiovascular centers stimulates the release of nitric oxide but also reduces hypertension induced by L-glutamate, aldosterone, or salt [4–6].

It is well known that the rostral ventrolateral medulla (RVLM) is a key region for control of sympathetic outflow and blood pressure [7]. Overactivity of sympathetic tone is a hallmark of cardiovascular disorders including hypertension and heart failure [8]. It is interesting that sympathetic activity is also increased in the ovariectomized (OVX) rats [9–11]. Increased oxidative stress is reported to be relative to hypertension development [12]. Oxidative stress results from an imbalance of generation over degradation of the reactive oxygen species (ROS), especially superoxide [13]. It is well known that NADPH oxidase (NADPHase) transfers electron to molecular oxygen and formats superoxide [14]. Superoxide dismutase 1 (SOD1) is enzyme that alternately catalyzes the dismutation of the superoxide radical into either ordinary molecular oxygen or hydrogen peroxide [15]. Therefore, ROS production is closely relative to activity of NADPHase and SOD1. The increased sympathetic outflow in hypertension is associated with enhanced oxidative stress at the level of RVLM [16]. Interestingly, administration of estrogen counteracts oxidative stress in erythrocytes and plasma of OVX rats and in premenopausal women [17, 18]. However, it is unclear if the beneficial effect of estrogen replacement on the OVX-induced sympathetic overactivity is associated with suppression of oxidative stress in the RVLM. Therefore, the present study was designed to determine the level of oxidative stress in the RVLM in the OVX rats and further evaluate the effect of estrogen replacement on oxidative stress.

## 2. Materials and Methods

### 2.1. Animals

Female Sprague-Dawley rats (Sino-British SIPPR/BK Laboratory Animal Ltd., Shanghai, China) were used in these experiments. All of the procedures of this study conformed to the institutional animal care guidelines and all performances were approved by the Animal Care and Use Committee of the Second Military Medical University. The rats were assigned to 4 groups: sham with vehicle (sham + vehicle), sham with 17*β*-estradiol injection (sham + E2), OVX with vehicle (OVX + vehicle), and OVX with 17*β*-estradiol injection (OVX + E2).

### 2.2. Ovariectomy

Ovariectomy was carried out according to previous study [19]. At 10 weeks of age, animals were anesthetized by isoflurane (induction 4%; maintenance 1.5%). The abdomen of the rat was cleaned and disinfected with 75% ethanol. An abdominal median incision was made and bilateral ovaries were removed. The rat that received the same operation without removing ovaries was regarded as the sham group. After surgery, the animals received intramuscular injection of antibiotics. One week after being OVX, rats were treated with 4-week subcutaneous injections of estrogen (17*β*-estradiol-water soluble, 30 *μ*g/kg/day, Sigma, St. Louis, MO, USA) [20] and 0.9% saline was used as vehicle treatment. Finally, uterine weight and serum samples were collected for assessing the effectiveness of OVX and estrogen treatment [21]. Serum sample was diluted by 1 : 100 and performed to detect estrogen concentration by estradiol Elisa kit (BioTNT Co.).

### 2.3. Measurement of Cardiovascular Parameters

The procedure for general surgery was described previously [22]. Briefly, rats were anaesthetized (urethane 800 mg/kg, alpha-chloralose 40 mg/kg, i.p.) and the trachea was cannulated. The right femoral artery was catheterized for BP measurement by the PowerLab system. The mean arterial pressure (MAP) and heart rate (HR) were derived from the BP pulse. Body temperature was kept at 37°C [23]. The renal sympathetic nerve was isolated retroperitoneally and placed on a pair of silver recording electrodes. The renal sympathetic nerve activity (RSNA) signal was amplified, integrated, and recorded with the PowerLab system (AD Instruments, Australia). The maximum nerve activity (Max) and background noise level of RSNA were obtained as described previously [24]. Briefly, Max occurred 1-2 min after the rat was euthanized with an overdose of pentobarbital sodium. Baseline RSNA, subtracting the noise level from the absolute value, was expressed as a percentage of Max.

### 2.4. Measurement of Norepinephrine (NE) Concentration

As described previously [22], the norepinephrine (NE) in 24-h urine was detected by High-Performance Liquid Chromatography (HPLC, Model 582 pump, ESA, USA) with electrochemical detection (Model 5300, ESA, USA). Briefly, urinary samples were collected by placing rats in metabolic cages for 24-h and embalmed with glacial acetic acid. The internal standard was dihydroxybenzylamine (DHBA; Sigma). NE was absorbed onto acid-washed alumina with 1.5 mmol/L tris HCl (pH = 8.8). Then we performed shaking of NE and standing for a while before being extracted with 0.2 mol/L glacial acetic acid (400 *μ*L). Supernatant was injected into HPLC column (reverse phase, ESA 150 × 3.2 mm, 3 *μ*m C18 (P/N 70-0636)), and NE was eluted with mobile phase. The flow rate was 0.4 mL/min. The experiments were performed at a temperature of 22–26°C.

### 2.5. Western Blot Analysis

The protein expression of NOX4 and SOD1 in the RVLM was detected by Western blot, as described previously [22]. Rats were euthanized by overdose of anesthetic and the brains were removed. The RVLM tissues were punched from 100 *μ*m coronal sections of brainstem according to the rat atlas [25]. The tissues were prepared and centrifuged. The total protein concentration was determined and equal amounts of protein (20 *μ*g) were applied to a 10% SDS-polyacrylamide gel, followed by transferring to PVDF membrane. The membrane was blocked and incubated overnight at 4°C with NOX4 antibody (1 : 2000, Epitomics, America) or SOD-1 (1 : 2000, Epitomics, American). The following day, the membrane was incubated with goat anti-rabbit IgG (H + L) for 2 h at room temperature. Finally, the membrane was visually detected and analyzed [26]. Tubulin was severed as loading control.

### 2.6. Measurement of ROS Production in the RVLM

In this study, two measurements were performed to detect the ROS production in the RVLM tissue. After RVLM tissue was punched and weighed from the rat which was euthanized (pentobarbital sodium, 300 mg/kg, i.p.), 80 *μ*L Protein Lysis Buffer (Cell Signaling Technology, USA) was added into the test tube and tissue was polished by electric homogenizer and then centrifuging for 20 min. Supernatant was collected for analysis by lucigenin chemiluminescence quantitative kit (Genmed Scientifics Inc., USA, GMS10113.5) and dihydroethidium (DHE). We can complete lucigenin chemiluminescence quantitative detecting according to the instructions. DHE, ROS sensitive fluorescent dye, brain tissues (15 *μ*m thick) were incubated at 37°C with DHE (5 *μ*mol/L) for 30 min. Sections were washed in 0.1 M PBS (3 × 1 min) and then examined by confocal laser scanning microscope (Fuji Film, Japan) and the image was captured at red fluorescence microscope around the RVLM and was evaluated using LAS-AF-Lite software [24].

### 2.7. Statistical Analysis

Data are presented as mean ± SEM. The difference of plasma estrogen concentration between sham-operated group, ovariectomized group, and ovariectomized rats with estrogen replacement group was analyzed by one-way ANOVA, followed by SNK post hoc analysis. The differences between sham-operated and ovariectomized rats with vehicle or estrogen treatment were analyzed by two-way ANOVA, followed by SNK post hoc analysis. *p* < 0.05 was considered significant.

## 3. Results

### 3.1. OVX Model Assessment

**Figure 1:**
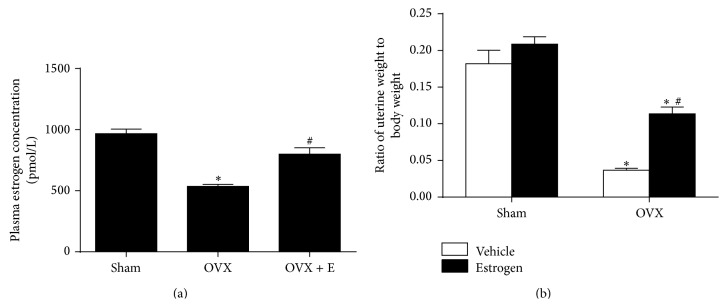
The plasma estrogen concentration as well as relative uterine weight in sham-operated, ovariectomized rats and ovariectomized rats with estrogen treatment for 4 weeks. (a) Plasma estrogen concentration; (b) uterine weight was attenuated body weight in sham and OVX rats with vehicle or estrogen treatment. Means ± SEM, *n* = 5/group, ^*∗*^ *p* < 0.05 versus sham group, and ^#^ *p* < 0.05 versus OVX or with vehicle group.

Compared with sham group, the OVX rats showed significant lower level in the relative uterine weight (0.182 ± 0.018 versus 0.037 ± 0.003 mg/g) and plasma estrogen concentration (966.7 ± 37.4 versus 535.8 ± 16.5 pmol/L), which was significantly attenuated by estrogen replacement (Figure 1).

### 3.2. The Cardiovascular Effect of Estrogen on OVX Rats

**Figure 2:**
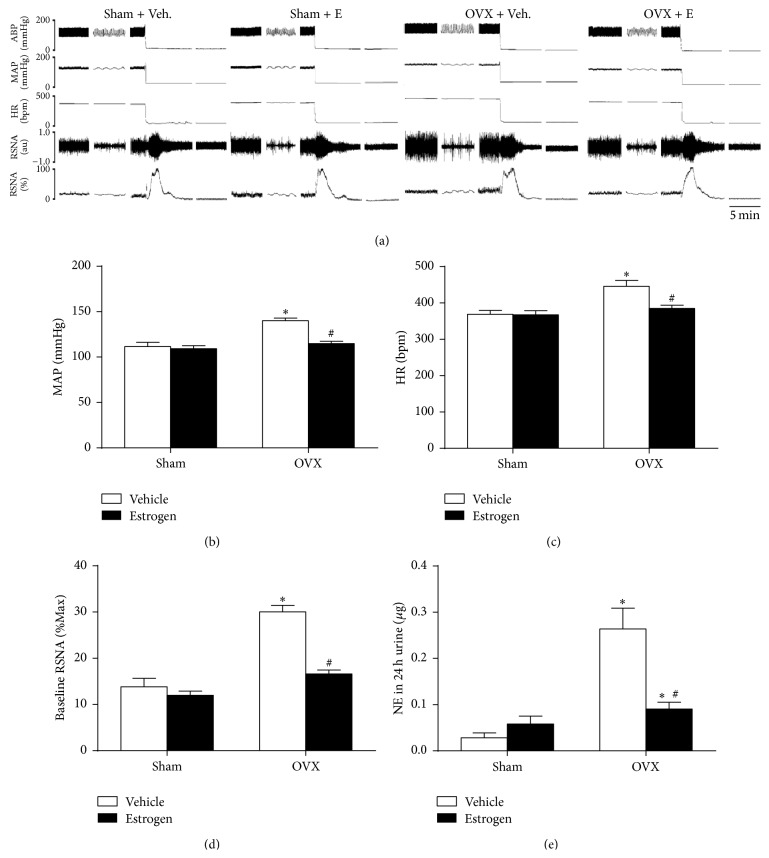
Cardiovascular changes in sham-operated and ovariectomized rats with vehicle or estrogen treatment for 4 weeks. (a) Representative original tracings of BP, HR, and RSNA in four groups. Maximum and background noise levels of RSNA were measured after rats were euthanized. Changes in MAP (b), HR (c), baseline RSNA (d), and NE in 24-h urine (e) were presented in four groups. Means ± SEM, *n* = 5/group, ^*∗*^ *p* < 0.05 versus sham + vehicle, and ^#^ *p* < 0.05 versus OVX + vehicle.

Levels of BP, HR, and RSNA began to be significantly increased 6 weeks after ovariectomy, which were completely prevented by subsequent injection of estrogen for 4 weeks (Figures 2(a)–2(d)). In additional, estrogen replacement also prevented the OVX-induced increase in NE in 24-h urine (Figure 2(e)).

### 3.3. Detection of ROS Production in the RVLM

**Figure 3:**
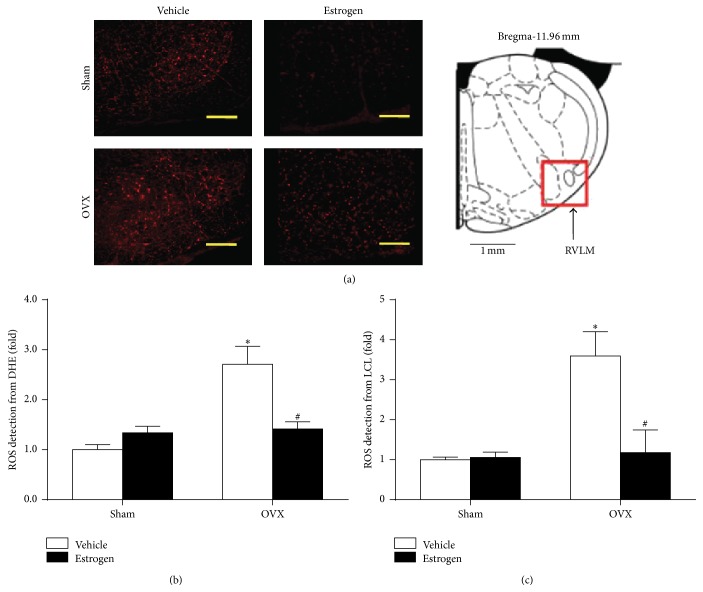
Estrogen attenuated the level of ROS in the RVLM of sham and OVX rats. (a) Representative confocal images of ROS (red) in the RVLM (indicated by a red square in the rat atlas) stained by fluorescent labeling (DHE). Scale bar, 200 *μ*m. Quantification of ROS production in the RVLM from DHE fluorescent analysis (b) and lucigenin chemiluminescence (LCL) detection (c). Means ± SEM, *n* = 5/group, ^*∗*^ *p* < 0.05 versus sham vehicle, and ^#^ *p* < 0.05 versus OVX with vehicle.

To elucidate the effect of estrogen on ROS production in the RVLM, fluorescent labeling (DHE) was used to detect ROS production, as indicated in Figure 3. The results of DHE fluorescent staining and lucigenin chemiluminescence quantitative detection showed that the level of ROS production in the RVLM was significantly higher in the OVX group than in sham group, which was reduced by estrogen replacement.

### 3.4. Protein Detection of NOX4 and SOD1 in the RVLM

**Figure 4:**
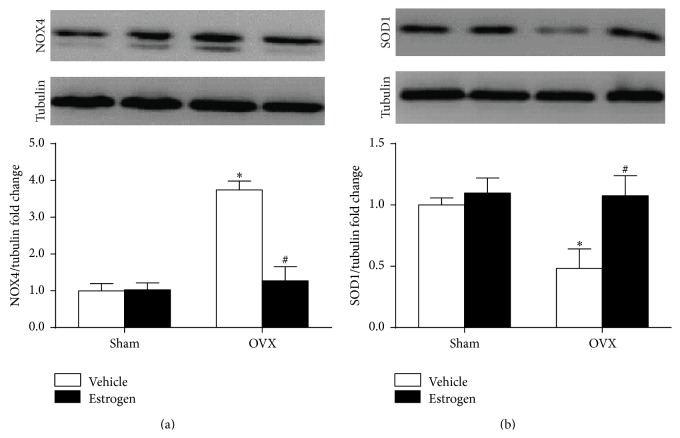
Representative bands (top) and quantification (bottom) of NOX4 (a) and SOD1 (b) in the RVLM of sham and OVX rats. Means ± SEM, *n* = 5/group; ^*∗*^ *p* < 0.05 versus sham + vehicle; ^#^ *p* < 0.05 versus OVX + vehicle.

As indicated in Figure 4, Western blot analysis demonstrated that ovariectomy procedure significantly increased and decreased NOX4 and SOD1 protein expression in the RVLM, respectively. It was found that changes in NOX4 and SOD1 protein expression in the RVLM of VOX rats were attenuated by estrogen treatment for 4 weeks.

## 4. Discussion

The main finding from this study is that OVX rats show a significant increase in BP and sympathetic activity as well as ROS production in the RVLM, which can be attenuated by estrogen replacement. These data suggest that estrogen replacement decreases BP and sympathoexcitation in OVX rats, which maybe resulted from the estrogen-mediated depression of oxidative stress in the RVLM.

Accumulating evidences indicate that estrogens exert protective effects on cardiovascular disorder through actions within the CNS [4, 27]. Menopause is a cardiovascular risk factor, which is mainly related to abrupt withdrawal of estrogen, and the lack of estrogen contributes to sympathoexcitation in both human and animal models [11, 28]. The mechanism by which estrogen withdrawal increases sympathetic outflow is not clear. Several regions in CNS, including the nucleus tractus solitarius, RVLM, and the paraventricular nucleus (PVN), are known to be involved in regulation of sympathetic tone and BP [29]. Abnormalities in the RVLM neurons contribute to sympathetic overactivity, which is associated with the development and progression of cardiovascular disorders including hypertension and chronic heart failure [30, 31]. It is reported that BP, HR, and NE (an index of sympathetic nerve activity) were increased in OVX rats [11]. In this study, it has been confirmed that, under anesthesia state, OVX rats show a significant increase in BP and RSNA, which can be attenuated by estrogen replacement. This is similar to that observed previously in conscience, freely moving OVX versus sham rats [32]. Therefore, these data lead to a conclusion that withdrawal of estrogen is a major contributor to sympathoexcitation in the OVX rats.

Sympathoexcitation is closely associated with the development and progression of cardiovascular diseases [30]. High level of oxidative stress in the RVLM which is resulting from abnormalities of renin angiotensin system and proinflammatory cytokines is responsible for increased sympathetic outflow [33, 34]. In this work, *β*-estradiol (water soluble) was used for estrogen replacement. This drug is water soluble and belongs to steroid hormone and penetrates the blood brain barrier, so it was applied by subcutaneous injection in this work. Although we did not detect the effective concentration of estrogen in the RVLM, this dosage of subcutaneous administration led to a significant reduction in ROS production at the level of RVLM and sympathetic outflow. Therefore, the concentration of estrogen has effect on RVLM neurons. Our findings have shown that the level of ROS in the RVLM is significantly increased in OVX rats, which is effectively prevented by estrogen replacement. Expression of NOX4 (NADPHase subtype) is increased and SOD1 is decreased in OVX rats compared with sham rats. NOX4 is predominantly involved in ROS regeneration among the NOX family in brain [35]. The antioxidant SOD catalyzes the dismutation of superoxide into hydrogen peroxide [36]. It is reported that overexpression of SOD in the RVLM attenuates the angiotensin II-induced oxidative stress [37]. Based on the present and previous work, we suggest that changes in NADPHase and SOD1 play an important role in high level of oxidative stress in the RVLM of OVX rats.

The more important finding in this work is that estrogen administration significantly reduced ROS production at the level of RVLM in OVX rats. This data supports the idea that the estrogen-mediated antioxidative stress contributes to decrease of BP and sympathoexcitation via the central mechanism. However, there are several limitations in this work. First, it is reported that serum estrogen and uterine weight were increased during prooestrus compared to dioestrus, but both of them in ovariectomized rats were decreased significantly compared with sham rats during either prooestrus or dioestrus [38]. Therefore, the success of OVX model was usually assessed by the levels of serum estrogen and relative uterine weight. In this previous study, the baseline of MAP presents fluctuation and baseline blood pressure is higher in dioestrus compared to prooestrus rats. The change difference of MAP caused by estrus cycle was an average of 7.4 mmHg in this previous study [38], which was significantly lower compared with the difference caused by ovariectomy in our work (an average of 28.5 mmHg). Moreover, there are no differences between dioestrus rats and prooestrus rats in baseline HR, lumbar, and splanchnic and renal sympathetic nerve activity. Although estrous cycle influences, at least partially, cardiovascular function, it has little impact on the conclusion of this study. Second, in addition to RVLM, the other regions such as paraventricular nucleus (PVN) also contribute to regulation of sympathetic tone [7]. Gingerich and Krukoff found that estrogen attenuated the L-glutamate-induced pressor response by microinjection into PVN mediated by ER*β* receptor [5]. Site-specific injections of siRNA-ERbeta into PVN augmented aldo-induced hypertension [6]. These evidences indicate that the other centers may play a role in mediating the effect of estrogen on sympathetic outflow. Thirdly, It is not clear which receptor type in the RVLM is involved in meditating the effect of estrogen in OVX rats. Estrogen exerts its physiological effects mainly via two estrogen receptor (ER) subtypes: intracellular receptors (including ER*α* and ER*β*) or membrane estrogen receptors (mERs). It is reported that both ER*α* and ER*β* are expressed in the CNS [39]. Importantly, cardiovascular effects induced by injection of estrogen into the RVLM can be prevented by the ER*β* antagonist but not ER*α* antagonist [4]. However, it is reported that ER*α* is centrally in the subfornical organ and is involved in the cardiovascular response to angiotensin II [40]. Thirdly, the mechanism by which estrogen regulates the ROS production in the RVLM is not further determined in this work. According to previous studies, the possible link between estrogen receptor and antioxidative stress has been indicated. For example, it is reported that treatment of OVX-SHR with conjugated equine estrogen (CEE) reduces ROS generation and NADPHase activity and enhances SOD and catalase expression in vascular and heart tissue [41, 42]. Moreover, several studies have demonstrated that estrogen is capable of regulating the transcript factor NF-KappaB, which is an important factor for regulating NADPHase expression [39, 43]. Therefore, it is possible that functional state of some transcript factor (e.g., NF-KappaB) associated with NOX4 and SOD is regulated by estrogen. In addition, the significance of estrogen-mediated antioxidative stress in protection against cardiovascular diseases needs to be further investigated. Whether estrogen could effectively reduce the incidence of hypertension and its complication in menopausal women still remains under debate. Although the beneficial effect of estrogen replacement on cardiovascular diseases in menopausal women is widely reported, it is also found that this treatment increases risk of stroke and invasive breast cancer [44].
